# Retinol-binding protein 4 and its potential roles in hypercholesterolemia revealed by proteomics

**DOI:** 10.17179/excli2015-478

**Published:** 2015-08-28

**Authors:** Watcharapong Jugnam-ang, Supitcha Pannengpetch, Patcharee Isarankura-Na-Ayudhya, Chadinee Thippakorn, Chartchalerm Isarankura-Na-Ayudhya, Ratana Lawung, Virapong Prachayasittiku

**Affiliations:** 1 Department of Clinical Microbiology and Applied Technology, Faculty of Medical Technology, Mahidol University, Bangkok 10700, Thailand; 2 Center for Research and Innovation, Faculty of Medical Technology, Mahidol University, Bangkok 10700, Thailand; 3 Department of Medical Technology, Faculty of Allied Health Science, Thammasat University, Pathumthani 12120, Thailand

**Keywords:** hypercholesterolemia, proteomics, retinol-binding protein, inflammation

## Abstract

Effects of hypercholesterolemia on alterations of serum proteins have not been fully elucidated. Herein, using two-dimensional gel electrophoresis (2-DE) in conjunction with LC-MS searching has successfully been carried out to investigate the change of protein expression profiles as consequences of raised blood cholesterol at different levels (normal group: total cholesterol 200 mg/dL; borderline high group: total cholesterol 200-239 mg/dL; and high group: total cholesterol ≥ 240 mg/dL) (n = 45). Results revealed that down-regulation of retinol-binding protein 4 (RBP4) (-2.26 fold), transthyretin (-1.25 fold) and gelsolin (-1.47 fold) was observed in the high group. Meanwhile, the other proteins such as haptoglobin, complement factor B and CD5 antigen-like protein were up-regulated upto +3.24, +1.96 and +2.04 fold, respectively. Confirmation by Western blotting revealed a significant reduction of RBP4 (approximately 50 %) in individual samples derived from the high group. Presumptive conclusion can be drawn that down-regulation of RBP4 might be attributable to the inflammation of adipocytes caused by the release of proinflammatory cytokines (e.g. tumor necrosis factor α and interleukin-1β) from adipose tissues. Moreover, the decrease of transthyretin might also be taken into accounts since it is known that the transthyretin usually forms complex with RBP4 to prevent glomerular filtration and excretion through the kidney. The suppressing effect on RBP4 should be potentiated by the increase of complement factor B and CD5 antigen-like protein, which rendered the adipose tissues to overwhelm the liberation of RBP4 to blood circulation by metabolic and inflammatory processes. Such inflammation could further modulate the induction of cytokine release (e.g. IL-6 and IL-1β), resulting in the synthesis of acute phase protein, in particular, haptoglobin and C-reactive proteins from hepatocytes. However, the mechanism of gelsolin reduction remains unclear. Among these differentially expressed proteins, the RBP4 has been proposed as a major linkage between hypercholesterolemia, adipose tissues, liver and kidney, which is believed to be a potential biomarker for metabolic and cardiovascular disorders associated with dyslipidemia in the future.

## Introduction

Hypercholesterolemia, defined as high cholesterol levels in the blood, is one of the major risk factors for development and progression of artherosclerosis, cardiovascular diseases and stroke (Aguilar and Fernandez, 2014[1]; Cui et al., 2012[14]; Tracy and Tracy, 1997[37]). The causes of hypercholesterolemia are associated with increased age, overweight, smoking, alcohol consumption, and family history (Cui et al., 2012[14]; Le et al., 2006[24]). Data from World Health Organization (WHO, 2015[39]) showed that mean value of blood cholesterol in Thailand of both genders was in a high range (5.1-5.6 mmol/L or 196.91-216.22 mg/dL) and prevalence of raised blood cholesterol was in between 50.0-59.9 % (WHO, 2015[39]). Although, the hypercholesterolemia is asymptomatic, elevation of blood cholesterol for a long time leads to artherosclerosis and other pathological conditions. These include induction of oxidative stress at the blood-brain barrier (Dias et al., 2014[16]), induction of angiogenesis and breast tumors (Pelton et al., 2014[30]), induction of adipose dysfunction (Aguilar and Fernandez, 2014[1]), accumulation of cholesterol in macrophages and other immune cells (Tall and Yvan-Charvet, 2015[36]). However, the underlying mechanisms of such consequences remain not fully understood.

Proteomics approach is very useful and powerful technology for detection and identification of protein expression in cells and biological samples (for recent review please see Larance and Lamond, 2015[23]; Zhang et al., 2013[43]). Such expression can be detected even under normal condition or disease progression. Therefore, proteomics has extensively been applied not only to discover new biomarker but also to gain more understanding on the underlying mechanism of several diseases e.g. diabetes, cancer, alzeimer's disease and cardiovascular diseases (Ge and Wang, 2012[18]; Sallam, 2015[33]; Scott et al., 2005[34]; Zurbig and Jahn, 2012[46]). For the latter, proteomics profilings of cardiovascular diseases and their related disorders have been studied for more than a decade. For circumstances, changes of distribution of transthyretin (TTR) forms in serum of acute myocardial infarction (AMI) have been detected (Cubedo et al., 2012[12]). Moreover, differential expression of retinol-binding protein 4 (RBP4) has been observed in acute new-onset AMI patients and in high-risk patients with heterozygous familial hypercholesterolemia (FH) (Cubedo et al., 2014[13]). Potential protein markers e.g. haptoglobin and serum amyloid A have been discovered for atherothrombotic ischemic stroke diagnosis (Brea et al., 2009[6]). In-depth molecular mechanism of protein alterations after simvastatin treatment was investigated in moderate hypercholesterolemic patients (Alonso-Orgaz et al., 2006[2]). However, notification has to be made that most of the studies were conducted at the disease states or after treatment. As a matter of fact, detection of abnormalities as early as possible prior to disease development and progression is particularly important for health prevention and promotion. Therefore, the present study aims at investigating the molecular mechanism and association between different levels of blood cholesterol and human serum proteins by proteomics and seeking for potential protein target that will be applied as early biomarkers for clinical diagnosis in the future.

## Materials and Methods

### Sample collection

Blood samples were collected as left-over specimens from Center of Medical Laboratory Services, Faculty of Medical Technology, Mahidol University. Based on the guidelines from the 2013 National Cholesterol Education Program Adult Treatment Panel (NCEP:ATP III), the criteria for selection of blood samples in this study are as follows: Group I normal blood parameters (total cholesterol 200 mg/dL; n = 15); Group II blood samples with borderline high of total cholesterol (total cholesterol 200-239 mg/dL; n = 15); Group III blood samples with high total cholesterol (total cholesterol ≥ 240 mg/dL; n = 15). Other blood parameters were in the normal ranges (Table 1(Tab. 1)). Serum samples were prepared from venous blood and centrifuged at 2,500 *g* for 15 min. Then, serum samples were placed into microtubes and stored at −80 °C until used.

### Proteomics study 

#### Sample Preparation

Pooled serum samples were generated (3 5 samples; total = 15 samples per group). To improve the performance of proteomics analysis, high abundant proteins in the serum sample (e.g. albumin and immunoglobulin) were removed by using ProteoPrep Immunoaffinity Albumin and IgG Depletion Kit (Sigma Aldrich, USA) in accordance with the manufacturer's instructions. The depleted serum samples were subsequently precipitated by using 2-D Clean-up kit (GE Healthcare, USA). Then, the protein pellets were dissolved in sample buffer containing 7 M urea, 2 M thiourea, 4 % CHAPS; freshly prepared by supplementation with 10 mg/ml dithiothreitol (DTT) and 10 µl/ml protease inhibitor cocktail.

##### Determination of protein concentration

The protein concentration was measured by the Bradford's method. The known concentrations (2-10 mg/ml) of bovine serum albumin (BSA) were applied for protein standard curve. Briefly, 800 µl of protein solution (798 µl of deionized water and 2 µl of depleted serum protein in lysis buffer) reacted with 200 µl of Bradford solution (Bio-Rad Laboratory, USA). The mixture of protein solution was incubated at room temperature for 10 min before determining the optical density at wavelength of 596 nm.

##### Two-Dimensional Gel Electrophoresis (2-DE)

Two-dimensional gel electrophoresis was carried out using 2-D Electrophoresis System (GE Healthcare, USA) as described previously (Isarankura-Na-Ayudhya et al., 2010[19]) with some modification. One hundred twenty micrograms of protein sample was mixed with 340 μl of rehydration buffer (8 M urea, 4 % CHAPS, 2 mM TBP, 0.001 % bromphenol blue and 65 mM dithiothreitol) containing 1 % 3-10 NL IPG buffer. The mixture was loaded onto 18-cm IPG strips with pH range of 3-10 non-linear of an isoelectric focusing system (IPGphore^TM^). Samples were run through steps of strip rehydration (20 °C, 12 h) and isoelectric focusing (500 volts for 1 h, 1,000 volts for 1 h, and 8,000 volts to reach 33,000 volt·h).

The maximum current was maintained at 50 mA per one strip. After the complete process was accomplished, the strip was equilibrated twice times (15 min each) in equilibration buffer (50 mM Tris pH 8.8, 6 M urea, 30 % glycerol, 2 % SDS, 0.03 % bromphenol blue) supplemented with 65 mM DTT and 135 mM iodoacetamide to allow the cysteine residues to be reduced and then carbamidomethylated. The strip was subjected to the second dimensional separation (Hoefer^TM^ DALT) using a SDS-polyacrylamide gel (12.5 %). Separation of protein was executed under the applied voltage of 10 volt per gel at 20 °C until the bromphenol blue dye front reached 0.5 cm from the bottom of the gel.

##### Gel staining

The gels were stained with colloidal Coomassie blue staining according to the standard recommendation. Briefly, 2-DE gels were agitated for overnight with Coomassie blue. The gels were then destained with milli-Q water until a clear background was observed.

##### Differential protein expression analysis

After staining, gel images were acquired using Image scanner III (GE Healthcare, USA). Differential analysis was performed by ImageMaster 2D Platinum version 7.0 (GE Healthcare, USA) software tool. These included spot intensity calibration, spot detection and background subtraction. Quantification of intensity of each spot was performed in term of spot volume (area intensity). The total spot volume normalization method was applied in which the percentage of each spot volume on a gel image is calculated relatively to the total volume of all spots on that image. Then, determination of differentially expressed proteins was conducted by comparing the ratio of % volume values with control. After complete analysis, these gels were kept at 4 °C until protein identification.

##### In-gel trypsin digestion for protein identification

For identification of protein spots, proteins were cleaved by using in-gel trypsin digestion procedure. First, the protein spots were excised from 2-DE gel and washed twice times with 100 µl of 50 % acetonitrile (ACN)/25 mM ammonium bicarbonate (NH_4_HCO_3_) at room temperature for 15 min. Then, the solvent was removed and 50- 100 µl of 100 % acetonitrile was added for 10 min or until all gels were in white. After that, the acetonitrile was removed and 10- 15 µl of diluted trypsin (diluted 0.1 mg/mL stock trypsin 1:10 into 25 mM ammonium bicarbonate) was added into each tube at 37 °C for 16-24 hrs. Next, the supernatants were removed to new tubes. Peptide were extracted twice times by adding 15-25 µl of 50 % acetonitrile, 5 % trifluoroacetic acid (TFA) to each tube containing gel slice for 15 min. Then, the extracted peptides were removed and combined with the supernatant in new tubes. Finally, the extracted peptides were dried in a vacuum centrifuge to dryness prior to further experiments.

##### Peptide identification by ESI-Ion trap-Mass spectrometry

Peptides were reconstituted in 10 µl of 0.1 % formic acid (FA) and identified by liquid chromatography-mass spectrometry (LC-MS/MS) consisting of a liquid chroma-tography part (Dionex Ultimate 3000, Thermo Scientific, USA) in combination with an electrospray ionization (ESI)/ion trap mass spectrometer (Model amazon SL, Bruker, Germany). The LC separation was performed on a reversed phase column (Hypersil GOLD 50 × 0.5 mm, 5 μm C18), protected by a guard column (Hypersil GOLD 30 × 0.5 mm, 5 μm C18), eluted at a flow rate of 100 μl/min under gradient conditions of 5-80 % B over 50 min. Mobile phase A consists of water/formic acid (99.9:0.1, v/v), and B consists of acetonitrile (100, v/v). Mass spectral data from 300 to 1500 m/z was collected in the positive ionization mode.

##### Database searching for protein identification

To identify protein, all MS/MS spectra recorded on tryptic peptides derived from each spot were searched against protein sequences from NCBInr and SwissProt databases using the MASCOT search program (www.matrixscience.com).

##### Western blotting

Expression of retinol-binding protein 4 was confirmed by Western blotting. In detail, 10 µg of serum protein (15 individual serum samples) were separated by 12 % (w/v) SDS-PAGE, and then blotted onto a nitrocellulose membrane. The blotting was performed for 1.5 hrs at 110 Volts. After blotting, the membrane was stained with Ponceau S to visualize the position of protein. Then, the membrane was removed Ponceau S and washed three times by mixture of Tris-buffered saline and Tween 20 (TBST) buffer for 5 min at room temperature. The membrane was soaked with blocking solution (5 % w/v non fat milk in TBST buffer) for 1 h at room temperature. The membrane was washed three times for 5 min in TBST buffer and incubated at 4 °C for overnight with 1:2000 dilution of anti-RBP4 antibody (Thermofisher Scientific, USA) in TBS buffer, and then washed three times for 5 min with TBST buffer. And, the membrane was incubated at room temperature for 2 hrs with a 1:10000 dilution of horseradish peroxidase conjugated goat anti-mouse IgG secondary antibody in TBS buffer. Excess antibody was washed three times for 5 min with TBST buffer before detection. The positive band was detected using an enhanced chemiluminescence (ECL) system. After immunodetection, the membranes were stained with Coomassie Blue as internal control for normalization. Differential proteins were analyzed by ImageQuant TL software (GE Healthcare, USA).

##### Statistical analysis

All data are expressd as mean ± standard deviation (SD). For group comparisons, data were determined by Analysis of variance (ANOVA). The strength of each association is presented as the regression coefficient with 95 % confidence interval and *P-*value. A *P*-value < 0.05 was considered statistically significant.

## Results

### Lipid parameters among groups of study

As shown in Table 1(Tab. 1), the mean values of total blood cholesterol were of 183, 220 and 265 mg/dL for the normal, borderline high and high groups, respectively. Notification has to be made that the levels of LDL-cholesterol in all samples of the high group exceeded that of the reference value (< 130 mg/dL) while some of the borderline high group remained in the normal range. On average, the levels of HDL-cholesterol in all samples were in the acceptable range (> 40 mg/dL). A wide distribution was found only on the triglyceride content in each group since the normal value should be less than 150 mg/dL. Other parameters were in the normal ranges.

### Master map of normal human serum

**Figure 1:**
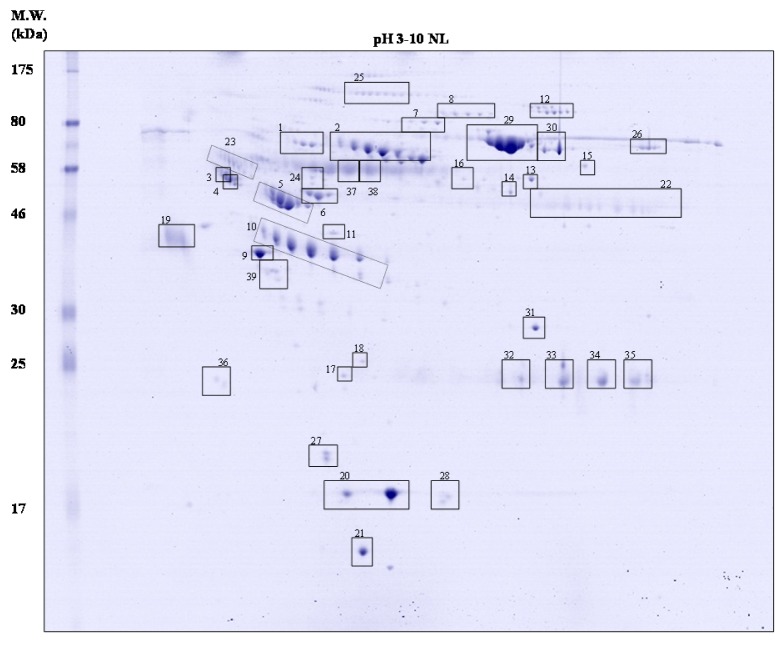
A master map of of human serum (after depletion of high abundance proteins) by 2-DE and Coomassie blue staining

Figure 1(Fig. 1) illustrates a master map of protein expression profile of human serum after removal of high abundant proteins (e.g. albumin and immunoglobulin). Approxi-mately 250 spots of protein were found on 18-cm 2-DE gel (with pH 3-10 non-linear gradient) stained with colloidal Coomassie blue. These proteins were further digested by trypsin and identified by electrospray ionization-Ion trap-mass spectrometry (ESI-Ion trap-MS) and the results were summarised in Table 2(Tab. 2).

### Comparative analysis of protein expression profiles in association with different levels of total cholesterol

**Figure 2:**
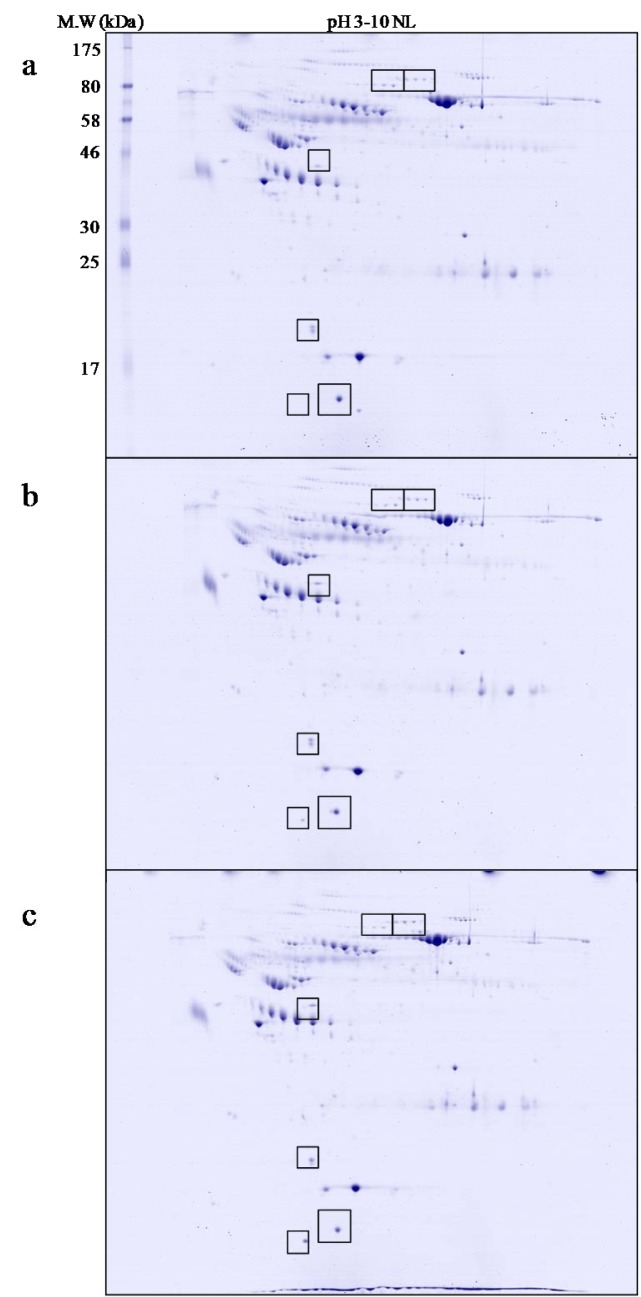
Comparison of 2-DE gels of human serum from normal (a), borderline high (b), and high total cholesterol (c) groups

**Figure 3:**
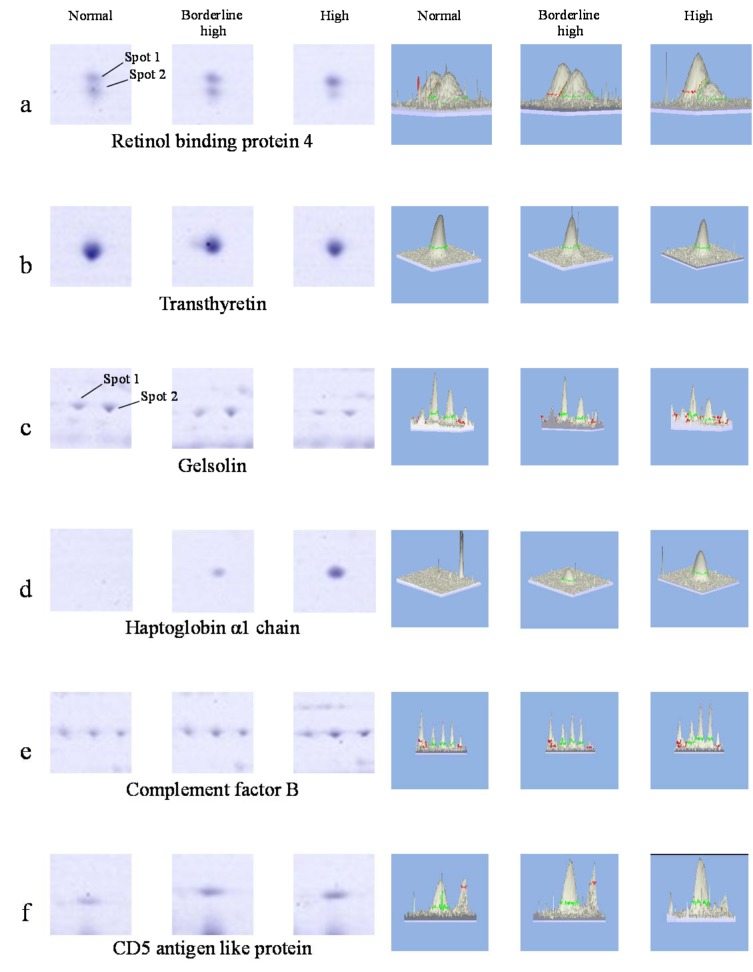
Down- and up-regulation of the changed proteins (a-f) found in normal, borderline high, and high total cholesterol groups

In comparative analysis, the total spots of protein were observed as 260 ± 13.08 spots, 243 ± 8.33 spots and 237 ± 8.89 spots in normal, borderline high and high groups, respectively (Figure 2(Fig. 2)). The proteomics profiles of serum in borderline high and high groups resembled those of the normal group. However, 6 groups of proteins (located in boxes) including retinol-binding protein 4 (spot no. 27 in Figure 1(Fig. 1) and Table 2(Tab. 2)), transthyretin (spot no. 21), gelsolin (spot no. 7), haptoglobin α 1 chain (absence in normal group), complement factor B (spot no. 8), 

CD5 antigen-like protein (spot no. 11) were differentially expressed. In zoomed gels, it is noteworthy that the retinol-binding protein 4 exists as two spots at the same *p*I (~5.7) with differences in molecular weight (designated as spots 1 and 2 for the higher and lower molecular weight, respectively) (Figure 3(Fig. 3)). The presence of these two isoforms was in consistency with previous studies (Cubedo, Padro, Cinca, Mata, Alonso and Badimon, 2014[13]). From our findings, three proteins (spot 2 of RBP4, transthyretin and gelsolin) were down-regulated while the others (spot 1 of RBP4, haptoglobin α 1 chain, complement factor B and CD5 antigen-like protein) were up-regulated when compared with those found in the normal group.

In quantitative study, the mean values of protein expression (represented in fold changes) from the pooled serum (3 5 samples; total = 15 samples per group) were extracted from 9 gels (3 independent experiments) (Table 3(Tab. 3)). Our findings revealed that the spot 2 of RBP4 was down-regulated upto -2.26 fold in the high cholesterol group while a lesser extent was detected in the borderline high group (-1.32 fold). The expression level of transthyretin was also reduced in borderline high group (-1.11 fold) and high group (-1.25 fold) when compared with the normal group. For the gelsolin, it was found that there were 2 isoforms separated by *p*I. Both isoforms were found to be decreased, particularly in the high group (1.36-1.47 fold). The expression level of complement factor B was higher in the borderline high group (+1.53 fold) and high group (+1.96 fold) than the normal group. Similar observation was also taken in the case of CD5 antigen-like protein (+1.83 fold and +2.04 fold in borderline high and high groups, respectively). Interestingly, the haptoglobin α1 chain was not detected in the normal group while high expression of protein (upto +3.24 fold) was observed in a concentration dependent manner with the level of cholesterol.

### Western blotting analysis of RBP4

**Figure 4:**
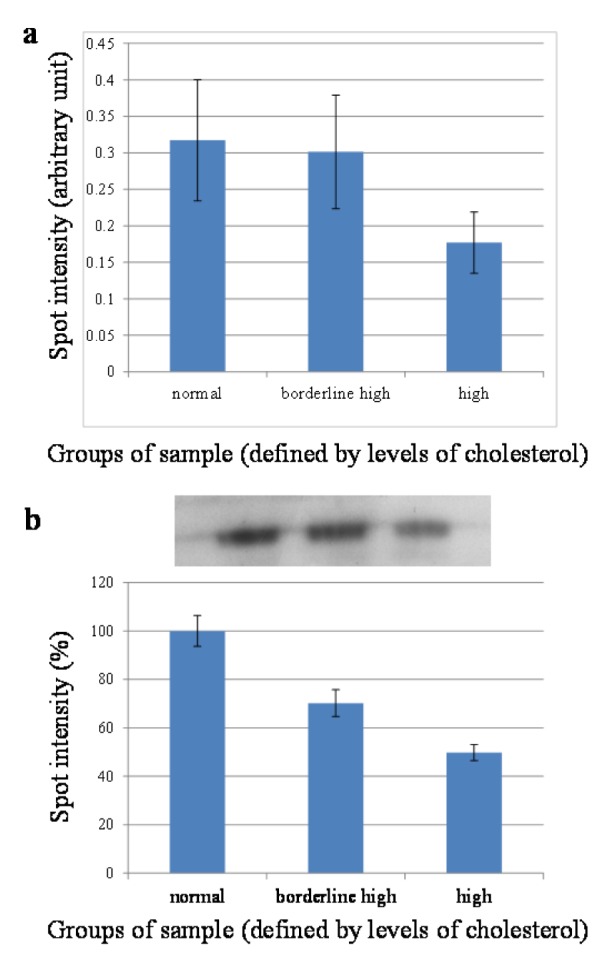
Determination of spot intensity of RBP4 by 2-DE (a) and Western blotting (b) in normal, borderline high, and high total cholesterol groups

To further confirm the expression level of retinol-binding protein 4 found in 2-DE, Western blotting was carried out on individual samples (n = 15). As shown in Figure 4b(Fig. 4), the bands of RBP4 located around 23 kDa in each lane. Results from the band intensity analysis (Figure 4b(Fig. 4)) indicated that the expression of RBP4 in the borderline high and high groups were of approximately 30 % and 50 % lower than that of the normal group, which were in good agreement with those observed on the 2-DE gels (Figure 4a(Fig. 4)).

## Discussion

**Figure 5:**
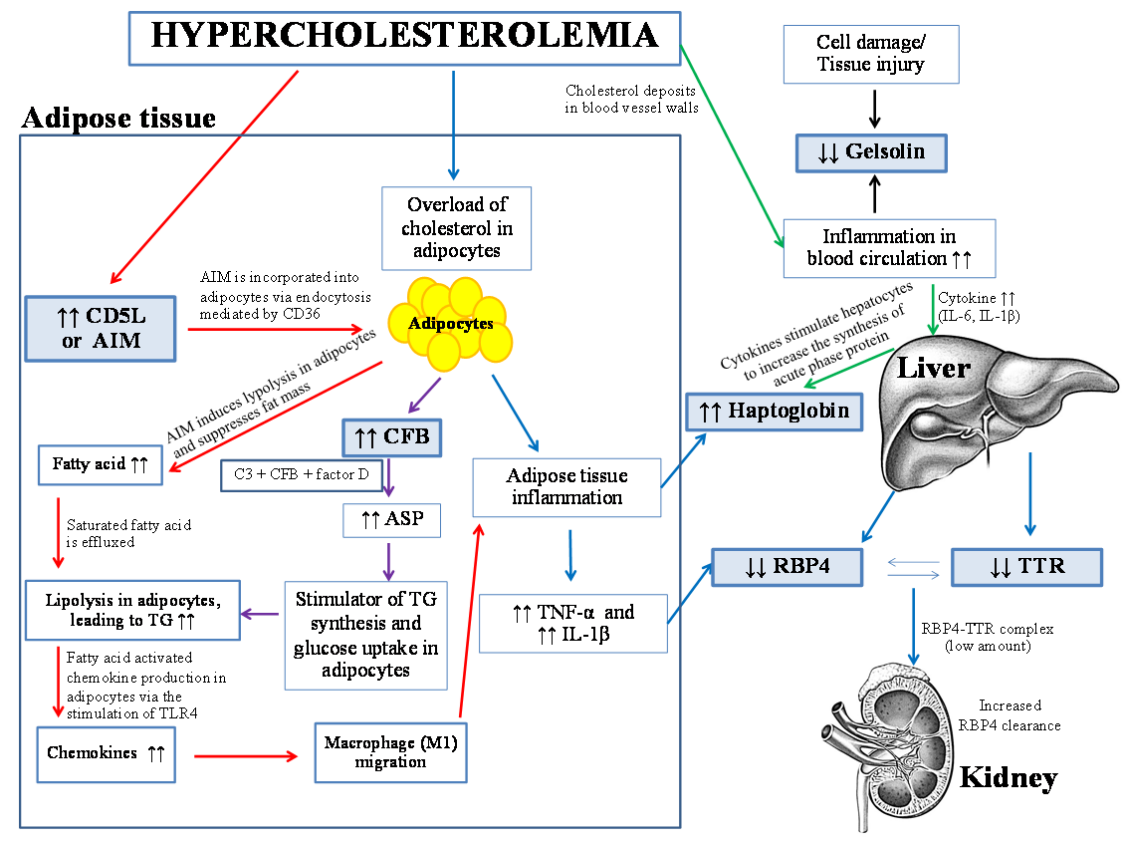
Plausible association of hypercholesterolemia and other human serum proteins and their roles in adipose tissues, liver, kidney and blood circulation

Herein, proteomics approach has successfully been applied to investigate the differential expression of serum protein under different levels of hypercholesterolemia. Results revealed that six protein spots from two-dimensional gel electrophoresis (2-DE) showed different expression levels in accordance with the levels of total cholesterol (Figures 2(Fig. 2) and 3(Fig. 3)). After protein identification by ESI-Ion trap-MS (Table 2(Tab. 2)), the function of these six proteins involved in lipid metabolism, inflammation, and immune responses. Three proteins, retinol-binding protein 4 (RBP4), transthyretin (TTR) and gelsolin, were lower in borderline high and high groups than that of the normal group (Figure 3a-c(Fig. 3), Table 3(Tab. 3)). While, expression of haptoglobin, complement factor B (CFB) and CD5 antigen-like protein (CD5L) were higher in borderline high and high groups than that of the normal group (Figure 3d-f(Fig. 3), Table 3(Tab. 3)). Plausible mechanisms of alterations of these serum proteins as consequences of hypercholesterolemia have been schematically depicted in Figure 5(Fig. 5) and summarised as follows.

First, our results revealed that the levels of RBP4 (spot 2) expression were negatively correlated with levels of blood cholesterol (Table 3(Tab. 3) and Figure 4(Fig. 4)). RBP4 is one of adipocytokines (synthesized from liver and adipose tissues) that delivers a retinol (vitamin A) from the liver into the tissue (Kotnik et al., 2011[20]). A decrease of RBP4 as a consequence of hypercholesterolemia might be attributable to the inflammation of adipose tissues. Previous studies suggested that adipose tissue is a major site of cholesterol metabolism. The existence of hypercholesterolemia is documented to induce adipocyte cholesterol overload and inflammation (Aguilar and Fernandez, 2014[1]). Such inflammation consequently led to the release of proinflammatory cytokines (e.g. tumor necrosis factor α (TNF-α) and interleukin-1β (IL-1β) from adipose tissues) (Coppack, 2001[11]; Zoccali et al., 2003[45]). In parallel, it is also reported that hypercholesterolemia and high cholesterol diet could increase the production of TNF-α and IL-1β, resulting in the amplification of inflammatory process (Kotnik et al., 2013[21]; Sell and Eckel, 2007[35]; Yoon et al., 2013[40]).

Supportive evidences can be drawn that hypercholesterolemia can increase the incidence of stroke and ischemic heart disease (IHD) as the effects of these proinflammatory cytokines (Cui et al., 2012[14]; Saito et al., 1996[32]; Zhan et al., 2014[42]). Accumulation of these proinflammatory cytokines further suppresses the release of RBP4 from adipose tissues into the blood circulation (Kotnik et al., 2013[21]; Sell and Eckel, 2007[35]). On one hand, it can be suggested that the level of RBP4 in the bloodstream was decreased by these proinflammatory cytokines. On the other hand, the decrease of serum RBP4 could also be affected by the inflammation of liver and the loss of RBP4 through the kidney. Our results revealed that the transthyretin (TTR) or prealbumin related with the levels of RBP4 (Figure 3(Fig. 3) and Table 3(Tab. 3)). TTR is a transport protein involved in the blood transport of different molecules with high binding capacity for thyroxine (T4), triiodothyronine (T3) and holo-retinol-binding protein. In the blood circulation, RBP4 forms a complex with TTR, which increases the molecular mass of RBP4 to prevent glomerular filtration and excretion through the kidney (Cubedo et al., 2012[12]). These evidences lend support to the decrease of RBP4 and TTR in human serum associated with hypercholesterolemia. The reduction of RBP4 in the blood circulation was also found in other pathological conditions such as acute myocardial infarction, ischemic heart disease, cholesterol gallstones, stroke, liver disease, and type I diabetes (Bahr et al., 2009[5]; Cubedo et al., 2014[13]; Pullakhandam et al., 2012[31]; Wang et al., 2010[38]).

Second, up-regulation of CD5 antigen-like protein (CD5L) and complement factor B (CFB) (approximately 2 fold) was found in the high group (Table 3(Tab. 3)). CD5L or apoptosis inhibitor of macrophage (AIM) is secreted by macrophages and has been shown to promote macrophage survival (Arai et al., 2005[4]). In this study, expression levels of CD5L in borderline high and high groups were higher than that of the normal group. This result is in a good agreement with other studies that found high expression of serum AIM in mice fed with high fat diet and AIM induction is involved in atherosclerogenesis by supporting the survival of macrophages within the lesions (Arai et al., 2005[4]; Kurokawa et al., 2010[22]). Blood AIM is incorporated into adipocytes via endocytosis mediated by CD36. Then, AIM induces lipolysis in adipocytes and suppresses increased fat mass. After that, saturated fatty acid is effluxed from adipocytes as a result of lipolysis. The increased fatty acid residues subsequently activated chemokine production in adipocytes via the stimulation of toll-like receptor 4 (TLR4). After chemokine induction, M1 macrophages are recruited into the adipose tissue by macrophage infiltration (Arai and Miyazaki, 2014[3]; Miyazaki et al., 2011[27]), resulting in the production of proinflammatory cytokines (TNF-α and IL-1β). This phenomenon was also potentiated by the increased expression of CFB. The CFB is centrally component of the alternative complement pathway. A cascade of alternative pathway activation consists of complement C3, CFB, and complement factor D. Previous studies have found that adipose tissues are the main source of these complements (Brocca et al., 2013[7]; Zhang et al., 2007[44]). They can produce acylation-stimulating protein (ASP) that is a stimulator of triglyceride synthesis and glucose transport in adipocyte and fibroblast cells. Furthermore, the complement system plays role in the initiation and maintainance of inflammation (Cianflone et al., 2003[10]; Zhang et al., 2007[44]). Taken together, the increases of CD5L and CFB rendered the adipose tissues to suppress the release of RBP4 by metabolic and inflammatory processes.

Third, expression of gelsolin and haptoglobin was in the opposite manner (-1.47 and +3.24 fold, respectively). Gelsolin is an intracellular actin-binding protein that involved in changes of cell shape, cell motility, and apoptosis. Gelsolin is produced by muscle that is an abundant protein of extracellular fluids capable of serving actin filaments and eliminating actin from the circulation (Yu et al., 2013[41]). Moreover, gelsolin binds and modulates the cellular effects of some bioactive lipids or inflammatory mediators such as lipopolysaccharide, lipoteichoic acid, lysophosphatidic acid, and platelet-activating factor (Osborn et al., 2007[28]). In this study, it is observed that elevated total cholesterol may in turn reduce gelsolin expression. To date, the mechanism of gelsolin in lipid metabolism and inflammation response remains unclear. Plausible explanation can be drawn that hypercholesterolemia causes the deposit of cholesterol in blood vessel walls and consequently induces inflammation in the blood circulation (Libby et al., 2000[26]). Supportive evidences on the decrease of gelsolin levels have been reported in acute injury and inflammation such as ischemic stroke, rheumatoid arthritis, chronic kidney disease, and infectious diseases (Peddada et al., 2012[29]). Some study has suggested that gelsolin may prevent inflammation and reduce blood clotting (Le et al., 2011[25]). Moreover, gelsolin is also associated with apoptosis that acts as anti-apoptosis by inhibition of cascade activities. In the case of haptoglobin, it is a glycoprotein produced by liver and adipose tissue. Therefore, the inflammation stated earlier led to the induction of cytokine release (e.g. IL-6 and IL-1β). These cytokines further stimulate the hepatocytes to increase the synthesis of acute phase protein, in particular, haptoglobin and C-reactive proteins (Castell et al., 1989[8]). Since it is reported that haptoglobin implicates in acute phase response to inflammation in which the high levels of haptoglobin were found in several diseases that involve in cardiovascular disease and inflammation such as overweight/obesity, artherothombotic ischemic stroke, and myocardial infarction (Chiellini et al., 2004[9]; De Pergola et al., 2007[15]). Other studies suggested that high levels of cholesterol were related with high inflammation sensitive plasma protein (ISP) including fibrinogen, haptoglobin, alpha1-antitrypsin, ceruloplasmin and orosomucoid (Engstrom et al., 2002[17]). In addition, inflammation is a major role in the development of artherosclerosis and cardiovascular diseases since it can reduce plaque stability and increase thrombogenesis that lead to CVD (Brea et al., 2009[6]; Engstrom et al., 2002[17]).

In summary, the molecular mechanisms of hypercholesterolemia-induced alterations of serum proteins were successfully explored by proteomics approach. Six proteins including retinol-binding protein 4 (RBP4), transthyretin (TTR), gelsolin, haptoglobin, complement factor B (CFB) and CD5 antigen-like protein (CD5L) were identified to play imperative roles in lipid metabolism and inflammatory processes as a consequence of hypercholesterolemia. Among these proteins, the RBP4 has been taken into accounts as a major linkage between hypercholesterolemia, adipose tissues, liver and kidney. Down-regulation of RBP4 expression or loss of RBP4 via the kidney clearance has been detected in all samples containing high blood cholesterol (> 240 mg/dL). Therefore, the RBP4 is believed to be a potential biomarker for metabolic and cardiovascular disorders associated with dyslipidemia in the near future.

## Notes

Chartchalerm Isarankura-Na-Ayudhya and Virapong Prachayasittiku (virapong.pra@mahidol.ac.th) contributed equally as corresponding authors.

## Conflict of interests

The authors have declared that no competing interests exist.

## Acknowledgements

This project is supported by the Office of the Higher Education Commission and Mahidol University under the National Research Universities Initiative and annual research budgets of Mahidol University (B.E. 2556-2558) and (B.E. 2557-2559). The authors would like to thank Mr. Pradit Panichanapun for his technical supports.

## Figures and Tables

**Table 1:**
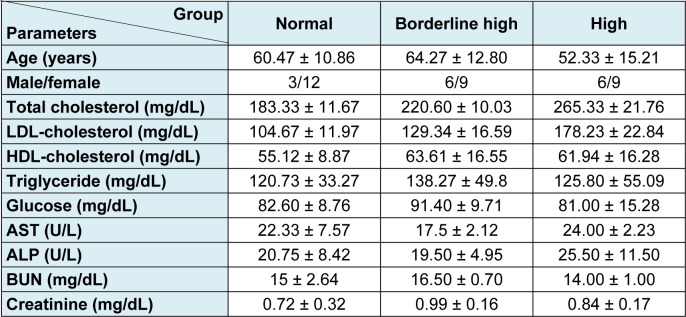
Characteristics and blood parameters of the studied group

**Table 2:**
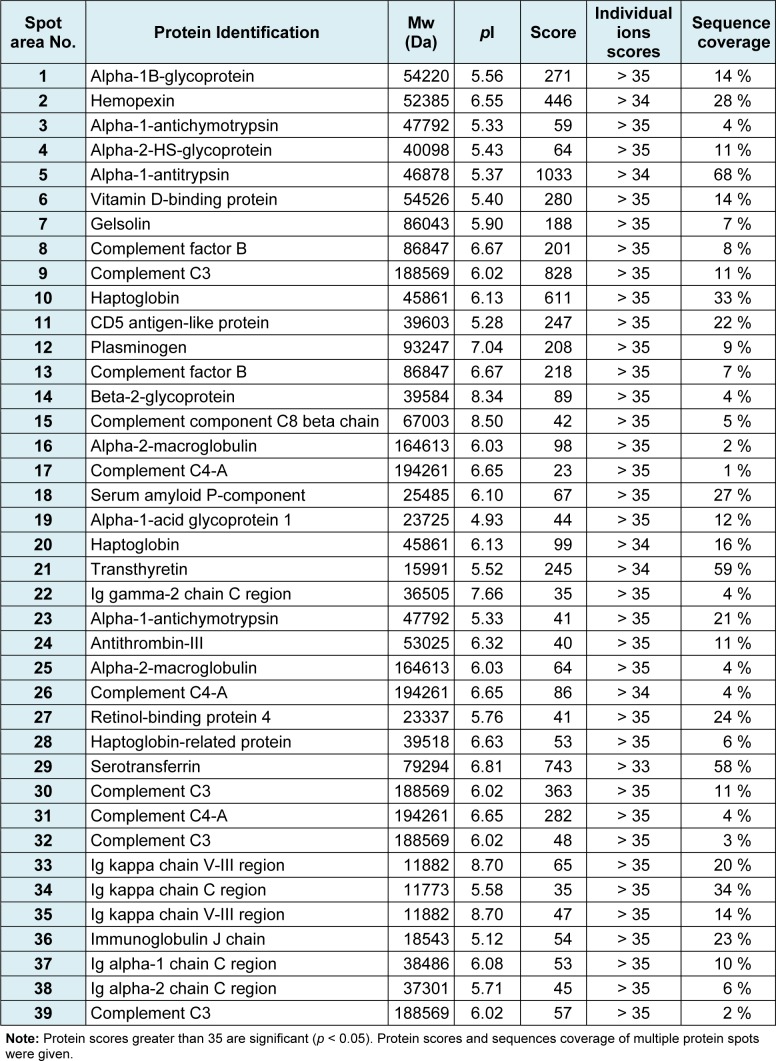
Peptide mass fingerprinting of human serum after depletion of high abundance proteins identified by ESI-Ion trap mass spectrometry

**Table 3:**
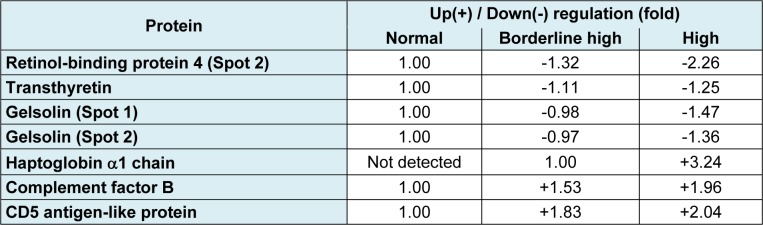
Changes of differentially expressed proteins of borderline high and high groups compared with normal group
